# QTL mapping for starch paste viscosity of rice (*Oryza sativa* L.) using chromosome segment substitution lines derived from two sequenced cultivars with the same *Wx* allele

**DOI:** 10.1186/s12864-021-07913-7

**Published:** 2021-08-05

**Authors:** Ling Zhao, Chunfang Zhao, Lihui Zhou, Qingyong Zhao, Zhen Zhu, Tao Chen, Shu Yao, Yadong Zhang, Cailin Wang

**Affiliations:** 1 Institute of Food Crops, Jiangsu Academy of Agricultural Sciences, Jiangsu High Quality Rice R&D Center, Nanjing Branch of China National Center for Rice Improvement, Nanjing, 210014 China; 2 Jiangsu Collaborative Innovation Center for Modern Crop Production, 210095 Nanjing, China

**Keywords:** Rice (*Oryza sativa* L.), Starch paste viscosity, Quantitative trait locus, Chromosome segment substitution lines

## Abstract

**Background:**

The eating and cooking qualities (ECQs) of rice (*Oryza sativa* L.) are key characteristics affecting variety adoption and market value. Starch viscosity profiles tested by a rapid visco analyzer (RVA) offer a direct measure of ECQs and represent the changes in viscosity associated with starch gelatinization. RVA profiles of rice are controlled by a complex genetic system and are also affected by the environment. Although *Waxy* (*Wx*) is the major gene controlling amylose content (AC) and ECQs, there are still other unknown genetic factors that affect ECQs.

**Results:**

Quantitative trait loci (QTLs) for starch paste viscosity in rice were analyzed using chromosome segment substitution lines (CSSLs) developed from the two cultivars 9311 and Nipponbare, which have same *Wx-b* allele. Thus, the effect of the major locus *Wx* was eliminated and the other locus associated with the RVA profile could be identified. QTLs for seven parameters of the starch RVA profile were tested over four years in Nanjing, China. A total of 310 QTLs were identified (from 1 to 55 QTLs per trait) and 136 QTLs were identified in more than one year. Among them, 6 QTLs were stalely detected in four years and 26 QTLs were detected in at least three years including 13 pleiotropic loci, controlling 2 to 6 RVA properties simultaneously. These stable QTL hotspots were co-located with several known starch synthesis-related genes (SSRGs). Sequence alignments showed that nucleotide and amino acid sequences of most SSRGs were different between the two parents. Finally, we detected stable QTLs associated with multiple starch viscosity traits near *Wx* itself, supporting the notion that additional QTLs near *Wx* control multiple characteristic values of starch viscosity.

**Conclusions:**

By eliminating the contribution from the major locus *Wx*, multiple QTLs associated with the RVA profile of rice were identified, several of which were stably detected over four years. The complexity of the genetic basis of rice starch viscosity traits might be due to their pleiotropic effects and the multiple QTL hot spots. Minor QTLs controlling starch viscosity traits were identified by using the chromosome segment substitution strategy. Allele polymorphism might be the reason that QTLs controlling RVA profile characteristics were detected in some known SSRG regions.

**Supplementary Information:**

The online version contains supplementary material available at 10.1186/s12864-021-07913-7.

## Background

Rice (*Oryza sativa* L.) is one of the most important cereal crops worldwide, with about half of the world’s population consuming it as a main source of dietary calories [1]. Grain quality is a key factor affecting rice production and market value. Current breeding programs aim to improve rice quality particularly with respect to eating and cooking qualities (ECQs).

As the main chemical component of grains, starch and its fine structure determine rice ECQs [2]. The starch viscosity profile is typically tested with a rapid visco analyzer (RVA) and offers a direct measure of ECQs by characterizing the pasting behavior and measuring the changes in viscosity associated with starch gelatinization during cooking. In recent years, starch RVA profiles have become an increasingly popular measure of rice quality, as they are easy to perform and only small samples are required [3]. Indeed, starch RVA profiles have been used as a novel tool to estimate ECQs, and some models were developed successfully in breeding programs [2, 4, 5].

Starch RVA profiles of rice grains are controlled by a complex genetic system that involves multiple quantitative trait loci (QTLs), several with major effects on the trait, and many starch synthesis-related genes (SSRGs) [6]. Linkage mapping using different populations identified a major QTL in the genomic region that contains the gene *Waxy* (*Wx*) on chromosome 6. *Wx* encodes the granule-bound starch synthase (GBSS) that is mainly responsible for longer amylose chains [7–9]. Many additional loci associated with starch RVA profiles were also shown to affect the ECQs of rice [10]. The alkali degeneration gene locus (*ALK*) on chromosome 6 encoding starch synthase II a (SSII-3), is the major factor responsible for gelatinization temperature (GT) and the distribution of amylopectin chain length [11–13]. Other SSRGs, such as genes encoding debranching enzyme (DBE), isoamylases (ISA), starch branching enzyme (SBE), soluble starch synthase (SSS), and pullulanase (PUL), play minor effects on starch RVA profiles [14]. These SSRGs have widely been proposed to control amylose content (AC), GT, or certain other rice quality traits [15–17].

The heredity of starch RVA profile characters is complex and could easily be effected by environmental factors, especially the temperature during the grain filling stage [18, 19]. This brings more difficulty in mapping QTLs of consistent RVA traits. Over 200 starch RVA traits-related QTLs have been identified in various mapping populations [3, 7, 18–25]. A few QTLs have been cloned or finely mapped (http://www.gramene.org/, http://www.ricedata.cn/). Certain varieties carrying the same *Wx* allele exhibit different ECQs, indicating that other minor QTLs affect ECQs. Identifying these minor QTLs will be critical to precisely modulate rice grain quality in the future. However, because the effects of minor QTLs might be covered by major QTLs such as *Wx*, fine mapping and the cloning of the underlying locus can be challenging. Populations derived from two parents with similar AC or with the same *Wx* allele have been developed to eliminate the effects of *Wx* and detect minor QTLs for starch RVA and ECQs of rice [23, 24, 26, 27].

The genetic basis of complex traits in rice and the contribution of single-nucleotide polymorphisms have been explored by genome-wide association studies, allowing the generation of testable hypotheses relating allele variation and starch RVA traits [28]. Allelic diversity of SSRGs can explain the genetic basis for the observed phenotypic diversity in starch physicochemical properties across germplasm [14, 17]. However, the distribution of functional alleles is strongly correlated with population structure, which can lead to false results if the population is small [29]. As one of the secondly populations, chromosome segment substitution lines (CSSLs) are very useful for precisely mapping QTLs and dissecting the genetic basis of complex traits [30]. A set of CSSLs had been developed which derived from two sequenced rice cultivars, the *indica* variety 9311 (recipient) and the *japonica* variety Nipponbare (donor) [31–33]. Since both cultivars carry the same *Wx-b* allele [22], this CSSLs could be used as an excellent mapping population to detect new QTLs that might be covered by the major *Wx* locus.

In this study, we aimed to detect stable QTLs in the absence of variation at the *Wx* locus for parameters of starch RVA profiles using CSSLs. Our results establish the foundation for fine mapping and subsequent cloning of genes responsible for these QTLs, which will help to improve ECQs in rice by molecular breeding.

## Results

### Performance of starch RVA profiles in parents and CSSLs in different years

The two parents 9311 and Nipponbare showed similar apparent amylose content (AAC), 15.5 % ± 0.2 and 14.6 % ± 0.2 %, respectively, in Nanjing from 2017 to 2018. The AAC of CSSLs ranged from 13.1 to 17.2 %, with an average AAC of 14.92 % in 2017. The starch RVA profiles of 9311 and Nipponbare differed significantly over four years of the study. Most RVA parameters varied greatly over four years, with the exception of peak time (PeT) and pasting temperature (PaT), which were less affected by environment (Table 1). For *indica* rice 9311, almost all parameters were much smaller than those in Nipponbare, with the exception of setback viscosity (SB) and PeT.

**Table 1:** Phenotypic variations of starch RNA characteristics among 9311, Nipponbare, and their CSSL populations

Traits		Parents		CSSLs			
	Year	Nipponbare	9311	Average	Range	Kurtosis	Skewness
PV/cP	2013	3,765.2±50.9	3,372.4±14.0	3573.4	3,003.0-4,074.0	0.98	–0.22
	2014	3,243.6±44.5	2,828.2±61.1	3046.4	2,610.0-3,863.0	2.4	0.47
	2016	2,978.5±29.2	2,197.9±22.6	1993.6	920.0-2,459.0	2.68	–1.48
	2017	3,372.3±38.7	2,162.5±76.2	1977.7	471.0-2,678.0	2.26	–1.28
TV/cP	2013	2,010.6±16.3	1,950.0±13.2	1965.2	1,719.0-2,189.0	–0.38	0.13
	2014	1,372.4±37.3	1,294.3±23.5	1490.5	1,232.0-1,992.0	1.09	0.92
	2016	1,423.0±25.7	1,290.5±24.1	1231.4	403.0-1,901.0	2.29	−0.73
	2017	2,373.3±124.2	1,520±141.2	1376.4	224.0-2,157.0	1.29–0.80	−0.80
FV/cP	2013	3,395.5±48.1	3,336.8±30.5	3381.6	3,077.0-3,702.0	–0.44	0.12
	2014	2,705.9±61.3	2,657.1±58.2	2859.2	2,477.0-3,280.0	–0.49	0.21
	2016	2,961.4±36.2	2,486.4±30.7	2283.0	978.0-2,954.0	4.29	–1.55
	2017	3,311.5±24.9	2,685.3±149.3	2550.4	668.0-3,562.0	3.35	–1.43
BD/cP	2013	1,755.4±28.6	1,422.1±37.2	1608.2	1,284.0-1,964.0	0.32	0.03
	2014	1,873.6±65.4	1,533.7±40.5	1547.8	1,024.0-1,973.0	0.06	–0.25
	2016	1,556.2±49.2	907.3±26.5	762.2	335.0-1116.0	0.23	–0.37
	2017	999.2±51.4	642.5±65.3	601.3	247.0-911.0	–0.31	–0.45
SB/cP	2013	–370.1±28.1	–35.6±5.5	–191.7	–604.0-225.0	0.17	0.14
	2014	–541.3±59.1	–170.5±30.6	–178.2	–659.0-489.0	–0.43	0.19
	2016	–17.6±6.7	288.8±12.3	289.5	–171.0-639.0	0.87	–0.18
	2017	–60.5±38.5	523.0±73.4	572.7	184.0-1060.0	1.05	0.13
PeT/min	2013	6.2±0.1	5.9±0.1	6	5.7-6.3	1.79	0.58
	2014	6.2±0.1	6.1±0.0	6.1	5.7-6.5	–0.51	0.12
	2016	6.5±0.1	6.2±0.1	6.2	5.5-6.8	0.78	–0.10
	2017	6.6±0.1	6.6±0.2	6.6	5.9-7.0	0.37	–0.35
PaT/°C	2013	74.7±0.9	86.3±3.1	81.9	73.6-87.1	–1.31	–0.69
	2014	76.7±0.5	74.4±0.6	77.9	72.0-88.6	–1.48	0.63
	2016	76.0±0.3	74.4±1.2	76.4	72.0-92.6	4.74	2.48
	2017	77.1±0.4	76.8±0.4	79.3	729.-94.9	0.92	1.65

**Fig. 1:**
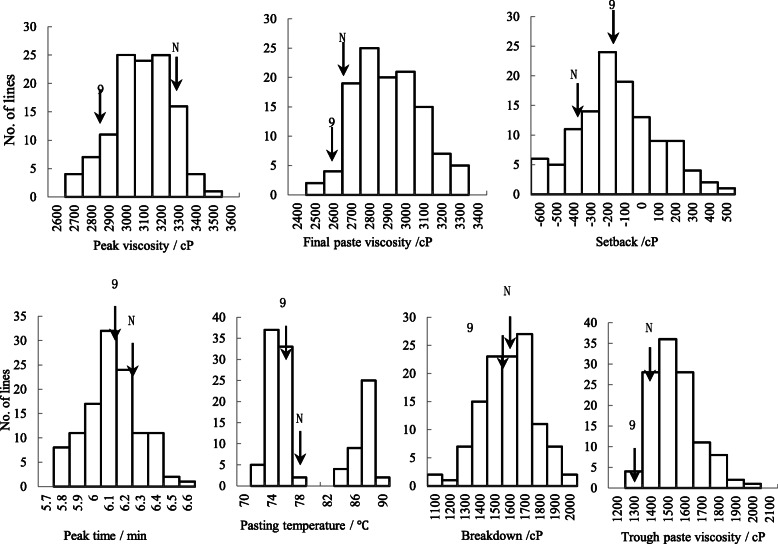
Distribution of starch rapid visco analyzer profile characteristics in the Nipponbare/9311 chromosome segment substitution line population in 2014. Note: The mean values for 9311 and Nipponbare are indicated by arrows labeled 9 and N, respectively

The starch RVA profiles showed a continuous distribution in the CSSL population over the different years, with the exception of PaT, which exhibited a double-peak distribution (Fig. 1). The maximum value of peak viscosity (PV) was greater than the other parameters as consequence of greater kurtosis. The starch RVA profiles of CSSLs showed a partial separation for some parameters, such as PV, final viscosity (FV), and PaT, in 2016 and 2017. Among the CSSLs, the phenotypic values of PV, FV, and PaT exhibited a greater range of variation than other traits over four years, whereas breakdown viscosity (BD), SB, and PeT varied little. The mean values for the various RVA parameters of the CSSLs were near the mid-parent value, but some values were not (Table 1). In addition, the phenotypic values for all starch RVA profiles showed bidirectional ultra-parental genetic types in the CSSL population, consistent with polygenic control of those quantitative traits.

Correlation analysis using average values for each year as a variable revealed that PV was positively correlated with trough viscosity (TV), BD, and FV, but negatively correlated with SB and PaT. TV was positively correlated with FV and PeT. FV was positively correlated with PeT and PaT but negatively correlated with SB. BD was negatively correlated with all other RVA parameters. SB was positively correlated with PeT and PaT. These correlations were highly significant (*P* < 0.01). Over four years, the relationship between SB and TV or FV changed (Supplementary Tables 1, 2, 3). SB was positively correlated with FV except in 2013. TV was positively correlated with SB in 2016 and 2017.

### Microclimate analysis

**Fig. 2:**
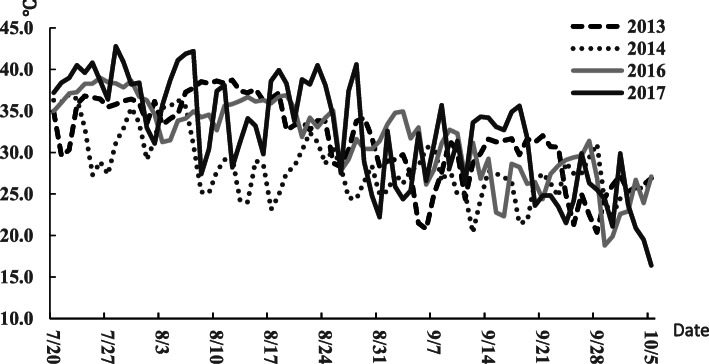
Daily maximum temperature recorded during the observational periods over the four years of this study

Every year, 9311 and Nipponbare bloomed on July 20th and August 25th, respectively. The heading time of CSSLs ranged from August 15th to the 25th. Although the maximum temperature of July and August was more than 40 °C in 2017, CSSLs and parents showed normal seed sets across the four years. The mean daily maximum temperature during the recorded periods ranged from 28.7 to 34.3 °C over the four years of this study. The daily average temperatures during observational periods in 2013, 2016, and 2017 were about 28.0 °C, higher than the average temperature of 25.1 °C measured in 2014 (Fig. 2). The average daily maximum temperatures showed the same tendency as the daily average temperatures over all four years. The highest recorded temperature of the four years was in 2017. The relative humidity (RH) varied extensively over four years independently of temperature. Average daily RH during the observational periods was higher in 2014 and 2017 at about 91 % lower than those in 2013 and 2016 at 80.5 and 82.5 %.

### QTL analysis

To elucidate the effects of environmental factors on starch viscosity among CSSLs, we mapped QTLs that influence starch RVA profile characteristics each year. In total, 310 QTLs were detected for seven RVA characteristics. Of them, 44 QTLs were related to PV, 42 to TV, and 43 to FV; 43, 49, 34, and 55 QTLs were identified for BD, SB, PeT, and PaT, respectively.

22 loci for PV, 15 for TV, 25 for FV, and 11 for BD were stably detected across multiple years. 28, 8, and 27 QTLs were mapped in different years for SB, PeT and PaT, respectively. Totally, 81 QTLs were mapped to the same genomic region across two years, and 50 QTLs were identified across three years. Five stable QTLs controlling TV, FV and PaT were identified across all four years (Table 2). The remaining 174 QTLs appeared in only one year. These results suggested that, although most traits showed large variation across the years, several QTLs related to starch RVA profile characters were robust enough to be consistently identified each year. For example, we repeatedly detected a QTL around 16.9–19 Mb of chromosome 2 that affects both FV and TV each year. Similarly, *qPaT7* and *qTV7* located near marker RM432 on chromosome 7 and *qFV9* located near RM219 on chromosome 9 were also mapped every year. A QTL near marker RM3827 on chromosome 6 for SB and a QTL near marker RM1812 on chromosome 11 for BD have not been previously described to be associated with these traits at the genomic locations (Table 3). Six QTLs were not affected by environmental factors in our research, and they are likely to substantially contribute to the RVA characteristics of rice starch.

**Table 2:** Quantitative trait locus (QTL) analysis for properties of the starch rapid visco analyzer profile

Trait	No. of QTLs				
	Mapped over two years	Mapped over three years	Mapped over four years	Total mapped QTLs	Repeatedly detected QTL (%)
PV/cP	18	4	0	44	50
TV/cP	10	3	3	42	35.7
FV/cP	13	10	2	43	58.1
BD/cP	3	8	0	43	25.6
SB/cP	15	13	0	49	57.1
PeT/min	5	3	0	34	23.5
PaT/°C	17	9	1	55	49.1
Total	81	50	6	310	43.9

**Table 3:** Stable rapid visco analyzer (RVA) quantitative trait loci (QTLs) detected more than three times

Chromosome	Marker	Interval (Mb)	Traits	Other RVA QTL co-located
1	RM488	25.75–27	SB	*qBDV1* [18]; *qBDV1* [24]
1	RM3143	28.05–29	BD	*qPKV1* [24]
2	RM5390	10.15–11.11	PaT, TV	*qCPV2, qSBV2* [34]; *qBDV2a, qCSV2a, qTD2* [23]
2	RM3795	16.9–20.95	FV^a^, TV^a^,SB, PaT	*qPKV2, qCPV2, qSBV2* [34]; *qPeT2* [15]
2	RM191	25.75–29.3	FV	*qBDV2* [35] ; *qHPV2* [34]; *qPaT* [27]
2	RM1342	28.65–29.3	PV, PaT	*qHPV2* [35]; *qPKV2* [18]
4	RM518	1.1–3.24	SB	*qPKV4* [27]
4	RM6748–RM5473	30.45–32.65	SB	*qPT4* [35]; *qASV* [36]
5	RM289	7.13–8.55	BD, PaT, SB, FV	*qSBV5, qCSV5* [34]; *qHPV5* [35]; *qPV5*, *qHPV5, qPeT5* [15]
5	RM178	24.59–26.37	BD, FV, SB ,PaT	*qPKV5, qHKV5* [22]
6	RM469–RM587	0.56–2.86	SB, PV, BD, PeT, PaT	*qPeT6* [3]; *qTPV6, qFPV6, qBDV6, qSBV6, qPKT6* [21]; *qHPV6–1, qCPV6, qCS6, qSBV6* [7]; *qPKV6, qBDV6, qSBV6, qPeT6* [24]; *qBD6, qSB6* [1]; *qTV6, qCPV6, qSBV6, qPeT6, qPKV6* [25]
6	RM527	9.31–10.98	SB, FV	*qPeT6* [24]
6	RM3827	22.55–23.8	SB	
6	RM3628	23.8–24.8	PV	*qHPV6* [1]
6	RM412	30.85–31.59	SB	*qBDV6, qCSV6, qCPV6, qHPV6, qSBV6* [18]
7	RM542	12.41–14.5	PeT	*qGT7* [23]
7	RM432	18.6–20.55	PaT^a^, TVa	*qCPV7* [18]; *qHPV7, qBDV7, qCPV7* [24]; *qGT7–1* [23]; *qPaT-7, qPeT-7* [25]
8	RM5485	23.32–25.4	FV	*qBDV8, qPeT8* [34]; *qPKV8* [24]; *qPKV8, qHPV8, qHPV6* [3]
9	RM219	7.39–9.11	BD, PV, SB, PaT, PeT, FV^a^	*qPaT9* [15]; q*PKV9,qBDV9-b* [25]
9	RM566	14.63–16.15	PV, PeT	*qPKV10* [34]; *qHPV9, qHPV9* [1]; *qCPV9, qCS9* [15]
10	10–1.63	0.8–2.12	BD, FV, SB, TV	*qCPV10, qSBV10, qCSV10* [3]; *qBDV10, qPeT10* [37]
10	10–9.12	8.76–9.57	TV^a^,FV, BD	*qPKV10, qHPV10, qCPV10, qSBV10, qPeT10, qCSV10* [3]; *qSB10* [27]
10	RM1375	15.89–18.04	BD	*qCPV10; qSBV10; qPeT10, qHPV10, qPKV10* [3]; *qPET10, qPAT10, qPKV10, qPKV10* [34]
11	RM1812	1.35–2.61	BD	*qBDV11, qPKV11,qPaT11* [25]
12	RM1261	15.52–18.07	FV	*qGT12* [23]; *qPaT12* [15] ; *qBDV12, qPaT12* [25]
12	RM1227	27.4–27.6	FV, SB	*qGT12* [23]

^a^The QTL was detected in each of the four years of the study

Several of the QTLs detected here exhibited pleiotropic effects, as they influenced multiple starch RVA profile characteristics. In total, 26 genomic intervals corresponding to 55 QTLs associated with starch RVA profiles were identified in at least three years. Of those, 13 chromosomal intervals showed pleiotropy by affecting more than one starch RVA profile characteristic (Table 3). In particular, the region near marker RM219 on chromosome 9 contained one QTL associated with all starch RVA profile characteristics with the exception of TV. Moreover, the interval between markers RM469 and RM587 on chromosome 6 was associated with five parameters, while the interval near marker RM3795 on chromosome 2, markers RM289 and RM178 on chromosome 5, and marker 10-1.63 on chromosome 10 were each associated with four RVA parameters.

Several reports have described QTLs affecting rice starch RVA profiles [18, 21–23, 34]. The 26 intervals detected more than three times were compared with previously mapped QTL. With the exception of two intervals, all other intervals co-located with known QTLs associated with starch RVA profiles from different mapping populations and different environments (Table 3). Specifically, a number of certain hot spots were identified, such as the region between markers RM469 and RM587 on chromosome 6 and the region around marker RM1375 on chromosome 10. In the genomic interval from markers RM469 to RM587 on chromosome 6, we mapped QTLs for SB, PV, BD, PeT, and PaT, as the previous studies did for the traits PeT, TV, FV, BD, and SB [1, 3, 7, 21, 24]. *qBD10*, located near RM1375 (15.9–18 Mb) on chromosome 10, overlapped with the mapping interval described for certain starch RVA profile QTLs related to FV, SB, PeT, TV, PaT, and PV [3, 24, 34].

We noticed that several of our stable QTL hotspots co-located with some known SSRGs, such as *Wx, SSIII-1*, *SSIV-2*, and *SBE3* (Table 4). Besides the QTL in the interval 0.56–2.86 Mb on chromosome 6, which co-located with *Wx*, the region spanning 3.24–5.38 Mb and 30.45–32.65 Mb on chromosome 4, 24.59–26.37 Mb on chromosome 5, and 16.9–20.95 Mb on chromosome 2 contained *PUL*, *SSIII-1*, *SSIV-2*, and *SBE3*, respectively. The loci *ADPlar (Adenosine diphosphate glucose pyrophosphorylase large subunit 1)*, *ADPsma (Adenosine diphosphate glucose pyrophosphorylase small subunit)*, *SSII-1*, and *ISA* were near our starch RVA QTLs. In the case of *ADPlar*, *ADPiso (Adenosine diphosphate glucose pyrophosphorylase large subunit 2)*, *GBSSII*, *SSII-2*, *SSIII-2*, *SSIV-1*, and *PUL*, the nearby or overlapping QTLs were identified in only one or two years, suggesting that the underlying QTLs are easily influenced by the environment. No QTLs associated with starch RVA traits was detected in regions that overlap with the genomic location of *SSI*, *SSII-3*, *SBE1*, or *SBE4*. The region between markers RM6748 and RM5473 on chromosome 4 contained a QTL for SB and overlapped with the genomic location of *SSIII-2*, which is related to SB. *SSIII-2* is a critical gene controlling Pat and PeT [6]. The effect of this region on Pat and PeT was not tested in our research.

**Table 4:** Co-location of known SSRGs with the starch rapid visco analyzer (RVA) quantitative trait loci (QTLs) detected in this study

Gene	Acc. number	Position (Mb)	Traits associated with QTL in this study	QTL interval (Mb)	Identity between 9311 and Nipponbare (%)	No. of Gap	No. of different aa
*ADPlar*	LOC_Os05g50380	Chr.5, 28.87	BD, FV	27.95–28.85	99.82	10	0
*ADPiso*	LOC_Os01g44220	Chr.1, 25.35	BD, PT, PeT	23.85–25.75	99.78	4	0
*ADPsma*	LOC_Os09g12660	Chr.9, 7.24	BD, PV, SB, PaT, PeT, FV^a^	7.39–9.11	99.75	9	5
*GBSSI (Wx)*	LOC_Os06g04200	Chr.6, 1.70	SB, PV, BD, PeT, PaT	0.56–2.86	100	0	0
*SSI*	LOC_Os06g06560	Chr.6, 3.08	–	–	99.94	7	0
*SSII-1*	LOC_Os10g30156	Chr.10, 15.67	BD98.06-100	15.89–18.04	99.64	8	4
*SSII-2*	LOC_Os02g51070	Chr.2, 31.23	PK, PK	30.45–34.75	99.66	3	4
*SSII-3*	LOC_Os06g12450	Chr.6, 6.75	–	–	100	0	0
*SSIII-1*	LOC_Os04g53310	Chr.4, 31.76	SB	30.45–32.65	99.81	24	4
*SSIII-2*	LOC_Os08g09230	Chr.8, 5.35	BD, FV, PK, TV	4.75–6.28	99.61	9	57
*SSIV-1*	LOC_Os01g52250	Chr.1, 30.04	PK, ST, TV	29.75–35.1	98.06	16	152
*SSIV-2*	LOC_Os05g45720	Chr.5, 26.48	BD, FV, SB ,PaT	24.59–26.37	99.7	10	0
*SBE1*	LOC_Os06g26234	Chr.6, 15.33	–	–	99.71	33	65
*SBE3*	LOC_Os02g32660	Chr.2, 19.36	FV^a^, TV^a^,SB, PaT	16.9–20.95	99.69	77	2
*SBE4*	LOC_Os04g33460	Chr.4, 20.12	–	–	99.87	15	21
*ISA*	LOC_Os08g40930	Chr.8, 25.89	FV	23.32–25.4	99.59	17	2
*PUL*	Os04G0164900	Chr.4, 4.40	BD, PeT, ST	3.24–5.38	74.69	402	78

^a^The QTL was detected in each of the four years

_ The QTL was detected only once or twice

The QTL hotspots near *SSII-1* and *SSII-3* were previously reported to have major effects on PT and minor effects on gel consistency (GC), AC, PV, CPV, BD, and SB [2]. Here, we found that the QTL near *SSII-1* is associated with BD. A QTL hotspot near *SBE3* on chromosome 2 was also reported, in which four major QTLs associated with HPV, consistency viscosity, viscosity at 95 °C, and BD over two years [27]. We also detected QTL hotspots that co-located with *SBE3* and are associated with FV, TV, SB, and PaT.

### Sequence alignment of SSRGs between parents 9311 and Nipponbare

The stable QTL hotspots were co-located with several known SSRGs. To explore the potential sequence variation between the two parental cultivars at these SSRGs, we aligned the nucleotide and predicted protein sequences from 9311 and Nipponbare, which revealed limited allelic diversity between the two parents, with some exceptions (Table 4). For example, the parental alleles for *SSI* and *SSII-3* were identical in sequence in 9311 and Nipponbare, possibly explaining why we failed to detect a QTL overlapping with either of these intervals in this study. Likewise, we detected no QTL near *SBE1* and *SBE4*, whose sequence identity between the two parents was 99.71 and 99.87 %, respectively.

The two parents also shared the same allele at *ADPlar* and *SSIV-2*, whose genomic coordinates did not overlap with any of our QTL intervals. However, our QTLs related to RVA profile parameters overlapped with or in close proximity to 12 SSRGs. Although the gene sequences of *Wx* are identical between 9311 and Nipponbare, we identified QTLs associated with multiple RVA traits in the *Wx* region. In addition, the nucleotide sequences and encoded protein sequences for *ADPsma*, *SSII-1*, *SSII-2*, *SSIII-1*, *SSIII-2*, *SSIV-1*, *SBE3*, *ISA*, and *PUL* were different between the two parents, suggesting that sequence polymorphisms at these loci contribute to the observed QTLs controlling RVA profile characteristics.

## Discussion

Recent studies have confirmed that changes in the environment largely affect rice starch PV, TV, and FV, while AC and other starch RVA parameters are mainly influenced by genotypes [1, 11]. In this study, starch RVA profiles of both parents and CSSLs varied greatly across four years, especially in 2016 and 2017. One possible reason is the high temperature of the grain filling stage during the summer of these years [38–40]. High temperature during flowering will make the spikelet sterile and reduce the seed set. Air temperature during grain filling significantly affects grain composition, as well as starch structure and properties [41, 42]. High temperatures result in lower AC, poor ECQs, and higher pasting properties by decreasing the activity of starch synthesis enzymes. Under such conditions, rice starch contained fewer long-chain amylose and short-chain amylopectin, but more intermediate- and long- chain amylopectin than under normal conditions, as well as higher crystallinity and gelatinization properties [42].

We observed that PV is positively correlated with TV, BD, and FV but negatively correlated with SB and PaT. Similar correlations were previously reported [1, 3, 34]. We also established that PV, FV, TV, BD, PaT, and PeT show consistent cross-correlations regardless of their great variations across the years, indicating that these traits are mainly affected by genotypic variation. There were main effect loci that controlled these starch RVA profile characteristics. The interaction between genotype and environment exhibited strongly for SB, as the correlation coefficients between SB and other parameters (except PV) differed for each year.

The confounding effects of the environment and the major effect gene *Wx* make it difficult to identify more minor QTLs for starch RVA profiles. Only a few genes and QTLs related to starch RVA profiles have been cloned, such as *qAC2*, *qGC6*, *ALK*, *Chalk5*, and *Dul* (*Dull*) [6]. In this study, we specifically selected two cultivars with the same *Wx* allele, *Wx-b*, to generate CSSLs as the mapping population [22]. *SSII-3*, the major gene controlling GT, showed no differences in its genomic sequence and only a 2-amino acid difference in its protein sequence between 9311 and Nipponbare. We therefore hypothesize that 9311 and Nipponbare carry the same allele at *SSII-3*. New QTLs that exert minor effects on starch RVA profiles and GT could be identified without the influence of major *Wx* and *SSII-3.* Zhang developed a CSSL population consisting of 38 lines with the same parents selected here. Following the same mapping strategy, 10 stable QTLs for RVA properties and two minor stable QTLs for GT were identified over two years and in two environments [22, 23].

The QTLs for the RVA properties they mapped also displayed pleiotropy but didn’t map to the same or similar chromosomal regions with our QTLs, which might not be surprising in considering that the two sets of CSSL lines harbored different chromosome segments from Nipponbare. Liu et al. (2011) also performed an extensive QTL mapping analysis for 16 rice quality traits across eight environments using a set of CSSLs developed from two rice varieties with similar AC, and detected 10 stable RVA profiles cross four environments [19]. However, the number of molecular markers was limited, raising the possibility of missing small introgression segments and thus lowering the accuracy of QTL detection. With more lines and smaller substitution segments, our CSSLs are adequate to identify minor QTLs for starch RVA profiles and GT without the effect of *SSII-3* and *Wx*.

In our research, although 310 QTLs were detected for seven paste viscosity properties for rice starch RVA profile traits, only 136 QTLs were mapped repeatedly. These results indicated that the RVA profile characteristics are largely affected by the environment, consistent with previous reports [18, 19]. The novel loci we detected for all starch RVA parameters, and in particular the six QTLs we identified every year, are important and require further validation. In addition, we propose that new and stable QTLs, such as *qSB6* and *qBD11*, which had not been previously reported, may be useful for research on marker-assisted selection of ECQs.

Based on our mapping, the clustering of multiple QTLs controlling RVA traits indicate that pleiotropic effects and QTL hotspots are key factors affecting starch RVA traits in rice. Similar observations of pleiotropy were reported previously (http://qtaro.abr.affrc.go.jp/, [3, 24]). QTLs with high correlations are often grouped in the same or adjacent marker intervals on a chromosome [43]. Starch RVA traits showed significant correlations that confirmed the linkage or pleiotropy of the corresponding loci.

Overlap between the intervals of our mapped QTLs and known SSRGs was common in this research (Table 4). Most known SSRGs showed different alleles between the parents, 9311, and Nipponbare. This result suggested that alleles of SSRGs contributed to our QTLs controlling RVA profile characteristics under the same major gene *Wx*, which should be taken into consideration in rice quality breeding. The effect of SSRG allele combinations on starch quality and ECQs should be investigated more in the future. We failed to identify QTLs associated with RVA profiles near some SSRGs, such as *SSII-3* and *SSI*, likely due to the lack of allele polymorphism between the two parents. The 5ʹ untranslated region (UTR), genomic and protein sequences of *Wx* are identical between 9311 and Nipponbare, although we noted several RVA traits associated with the *Wx* genomic region. Xu also reported several SNPs close to *Wx* that were significantly associated with RVA parameters in subpopulations with the same *Wx* allele [15]. These loci will be high-priority candidates for future characterization.

The highly consistent genotypes between SSRGs and QTLs reported here confirmed the accuracy of our mapping. The stable or newly developed QTLs located in the region that no known SSRGs in are meaningful to identify new genes controlling starch RVA profiles in the future, which will facilitate further research into the genetic mechanism regulating RVA profiles and ECQ of rice.

## Conclusions

We mapped QTLs associated with starch viscosity profile, one of the most important factors contributing to ECQs in rice. The effect of major locus *Wx* could be eliminated by generating CSSLs developed from 9311 and Nipponbare with the same *Wx-b* allele, which allowed the identification of new QTLs associated with RVA profile traits. We analyzed the genetic basis of variation in RVA profile over four years, and identified 136 repeated QTLs. Among them, 6 stable QTLs were detected every year, which are therefore likely to be very important for the RVA characteristics. In addition, we uncovered 13 intervals detected for 3 of the four years that showed pleiotropy with respect to controlling two to six starch RVA profile properties simultaneously. Finally, we highlighted four intervals, such as the interval between markers RM469 and RM587 on chromosome 6, that are associated with more than four RVA parameters for a given year. Pleiotropic effects and QTL hotspots appear to be key factors affecting starch RVA traits in rice. There was high consistency between allelic diversity at known SSRGs and the QTLs reported here. Future research will explore these stable QTLs and hotspots in more detail.

## Methods

### Plant materials and field planting

An advanced backcross population was developed by our lab using the *indica* variety ‘9311’ (recipient) and the *japonica* variety ‘Nipponbare’ (donor). 9311 was obtained from its breeder, Yangzhou Institute of Agricultural Sciences, Jiangsu Academy of Agricultural Sciences. Nipponbare was obtained from Jiangsu Provincial Platform for Conservation and Utilization of Agricultural Germplasm.

The backcrossed population consisted of 119 BC_4_F_2_ lines. Backcrossing and simple sequence repeat marker selection were performed as described in detail by Zhu and Zhao [20, 31]. Each introgression line in the population contained one to seven segments originating from Nipponbare. The 119 lines contained 318 substituted segments with an average of two to seven segments per line and covered 84.0 % of the whole rice genome [31].

The CSSLs and two parents were planted in fields at the Jiangsu Academy of Agricultural Sciences (32°02’N, 118°52’E; elev. 10 m) in Nanjing in 2013, 2014, 2016, and 2017. Each year, all seeds were planted within two blocks on May 15th and transplanted on June 20th. Each line was planted in three rows with a row-to-row distance of 30 cm and plant-to-plant distance of 13.3 cm. The seeds of five plants were sampled from each CSSL line and dried naturally. Milled rice was grinded into powder and then passed through a 100-mesh sieve. After drying at 4 °C in an oven, each powder sample was balanced for 2 days at room temperature and kept at 4 °C for 3 months. Then, paste viscosity was measured as described below. For each line, five samples were used to determine the starch RVA profile.

### Microclimate

The climate parameters at the field site (air temperature, RH) were measured using a data logger (Thermo Recorder TR-72U, T & D Corp, Japan). The sensor was placed at a height of 170 cm; air temperature and RH were collected every 10 min. The climate parameters were collected from the beginning of flowering to full maturity each year.

### Starch RVA profile

Starch paste viscosity was measured with a Rapid Visco Analyser (Tecmaster, Perten, Sweden) according to the American Association of Cereal Chemists Standard Method (AACC 61 − 02) with TCW software 3 (Thermal Cycle for Windows) [44]. 3 g rice flour (accounting for 12 % moisture basis) with 25 mL distilled water were used. The heat profile was set as follows: (1) the temperature was held at 50 °C for 1 min; (2) the temperature was linearly ramped up to 95 °C over 3.75 min; (3) the temperature was held at 95 °C over 2.5 min; (4) the temperature was ramped down linearly to 50 °C for 3.75 min; (5) the temperature was held at 50 °C for 1.4 min. The RVA paddle speed was set to 960 rpm for the first 10 s of the test, after which the speed was 160 rpm.

Starch paste viscosity characteristics are characterized by five parameters : PV, TV, FV, PeT, and PaT. BD and SB were calculated as: BD = PV – TV, and SB = FV – PV [36]. Correlations between the RVA parameters for each year were analyzed by IBM SPSS Statistics v22.

The AAC was determined using the iodine staining method described in the European Standard EN ISO 6647-2-2015. The absorbance of the solution was measured at 620 nm against the blank solution using a continuous flow analyzer (Seal Analytical AA3, GER). The AAC was calculated using a standard curve made from four rice samples with known AAC.

### QTL mapping

Genotype data for 250 polymorphic loci, including 211 simple sequence repeat and 39 sequence tag site markers, were used for QTL detection. Molecular linkages were established using composite interval mapping with version 3.3 of QTL IciMapping software [45].

QTL were detected according to the method described by Eshed and Zamir [46]. The significance of each QTL was determined by comparing the mean RVA profile values of a CSSL line with the recipient parent 9311 using analysis of variance and Dunnett’s test. A QTL was considered as present when a CSSL line exhibited a significant difference compared to 9311 with corresponding probability value *P* < 0.05. If more than three CSSLs showed differences, then the QTL was estimated as being located within the chromosomal region shared by those CSSLs [22]. QTL nomenclature followed as that of McCouch et al. [47].

### Sequence alignment of SSRGs between parents 9311 and Nipponbare

The genomic and predicted protein sequences of known SSRGs that co-locate with starch RVA QTLs were downloaded from online resources, as 9311 and Nipponbare genomes are sequenced. Nipponbare sequences were downloaded from Gramene (http://www.gramene.org/). The genomic sequences for 9311 were obtained from the Rice Genome Project (Beijing Genomics Institute) [32]. The predicted protein sequences were obtained from Gramene or NCBI (https://www.ncbi.nlm.nih.gov/). Sequence alignment was performed with DNAMAN (version 6).

## Supplementary Information

**Additional file 1: Table S1.** Correlation analysis of seven RVA parameters in 2013 and 2014. **Table S2.** Correlation analysis of seven RVA parameters in 2016 and 2017. **Table S3.** Correlation analysis of starch RVA parameters.

**Additional file 2: Table S4.** Genotype and phynotype of CSSLs.

**Additional file 3.** Sequence Alignments of SSRGs between 9311 and Nipponbare.

## Data Availability

All materials analyzed during the current study are available from the Jiangsu Provincial Platform for Conservation and Utilization of Agricultural Germplasm (http://jagis.jaas.ac.cn/CL_crop.aspx). The stock numbers for 9311 and Nipponbare are M3A00407620 and SD_NJAU_B149, respectively. The results of correlation analysis among RVA parameters in different years are in the Additional file (1) The stock numbers, raw phenotype data and genotype data for all CSSLs are in Additional file (2) The details about the sequence alignments are in the additional file 3.

## Abbreviations

ECQs: Eating and cooking qualities
RVA: Rapid Visco Analyzer
AC: Amylose content
QTL: Quantitative trait loci
CSSLs: Chromosome segment substitution lines
SSRG: Starch synthesis related gene
GBSS: Granule-bound starch synthase
ALK: Alkali degeneration
SS: Starch synthase
GT: Gelatinization temperature
DBE: Debranching enzyme
ISA: Isoamylases
SBE: Starch branching enzyme
SSS: Soluble starch synthase
PUL: Pullulanase
AAC: Apparent amylose content
PaT: Pasting temperature
PeT: Peak time
SB: Setback viscosity
PV: Peak viscosity
FV: Final viscosity
BD: Breakdown viscosity
TV: Trough viscosity
RH: Relative humidity
ADPlar: Adenosine diphosphate glucose pyrophosphorylase large subunit 1
ADPsma: Adenosine diphosphate glucose pyrophosphorylase small subunit
ADPiso: Adenosine diphosphate glucose pyrophosphorylase large subunit 2
GC: Gel consistency
Dul: Dull
UTR: Untranslated region
