# Supplementary material for: QTL mapping for starch paste viscosity of rice (Oryza sativa L.) using chromosome segment substitution lines derived from two sequenced cultivars with the same Wx allele
Source: BMC Genomics. 2021 Aug 5;22:596. doi: 10.1186/s12864-021-07913-7 (PMC8340499; doi:10.1186/s12864-021-07913-7)
Supplement: Supplementary file 3 — Additional file 3. Sequence Alignments of SSRGs between 9311 and Nipponbare. [file 12864_2021_7913_MOESM3_ESM.docx]

**Additional file 3. Sequence Alignments of SSRGs between 9311 and Nipponbare**

1. ***ADPlar***

The sequences of *ADPlar* were downloaded from <http://www.gramene.org/>. *LOC_Os05g50380* and *BGIOSGA017490* are the Gene IDs of Nipponbare and 9311, respectively. The same as below.

Result of DNA sequence alignment

* 9 indicates 9311 and N indicates Nipponbare, the same as below.

Result of protein sequence alignment

1. ***ADPiso*** (<http://www.gramene.org/>, LOC_Os01g44220 and *BGIOSGA004052*)

Result of DNA sequence alignment

Result of protein sequence alignment

1. ***ADPsma*** ([http://www.gramene.org/,](http://www.gramene.org/,%20LOC_Os09g12660) *LOC_Os09g12660* and *BGIOSGA030039*)

Result of DNA sequence alignment

Result of protein sequence alignment

1. ***GBSSI (Wx)*** ([http://www.gramene.org/,](http://www.gramene.org/,%20LOC_Os09g12660) *LOC_Os06g04200* and *BGIOSGA022241*)

Result of DNA sequence alignment

Result of 5’UTR sequence alignment

Result of protein sequence alignment

1. ***SSI*** (http://www.gramene.org/, *LOC_Os06g06560* and *BGIOSGA021860*)

Result of DNA sequence alignment

**

Result of protein sequence alignment

1. ***SSII–1***

Result of DNA sequence alignment (<http://www.gramene.org/>, *LOC_Os10g30156* and *BGIOSGA033011*)

Result of protein sequence alignment (<https://www.ncbi.nlm.nih.gov/protein/>, XP_015614561.1 and ACY56180)

1. ***SSII–2*** (<http://www.gramene.org/>, *LOC_Os02g51070* and *BGIOSGA005631*)

Result of DNA sequence alignment

Result of protein sequence alignment

1. ***SSII–3*** (<http://www.gramene.org/>, *LOC_Os06g12450* and *BGIOSGA022586*)

Result of DNA sequence alignment

Result of 5’UTR sequence alignment

Result of protein sequence alignment

1. ***SSIII–1*** (<http://www.gramene.org/>,*LOC_Os04g53310* and *BGIOSGA014316*)

Result of DNA sequence alignment


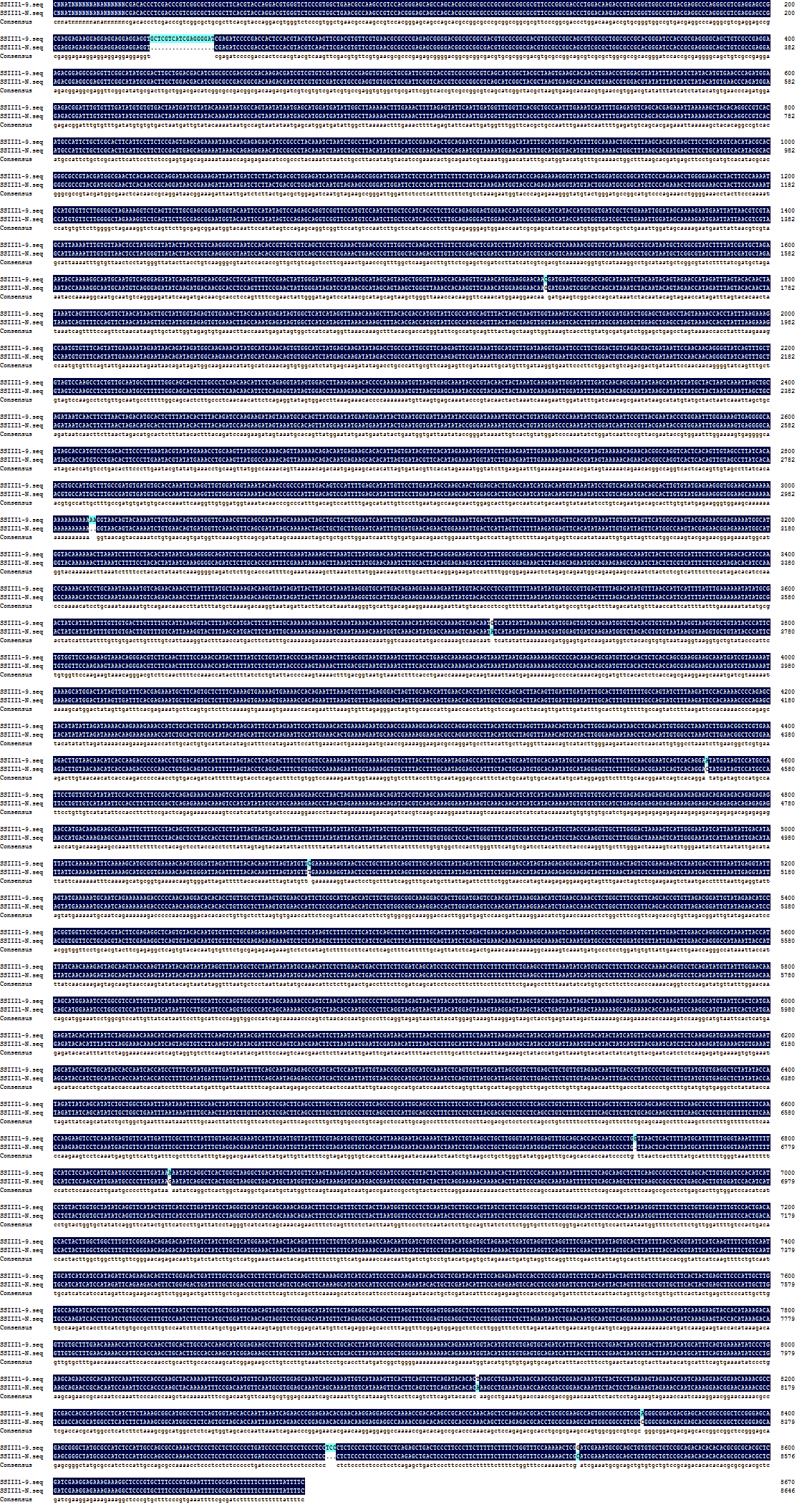


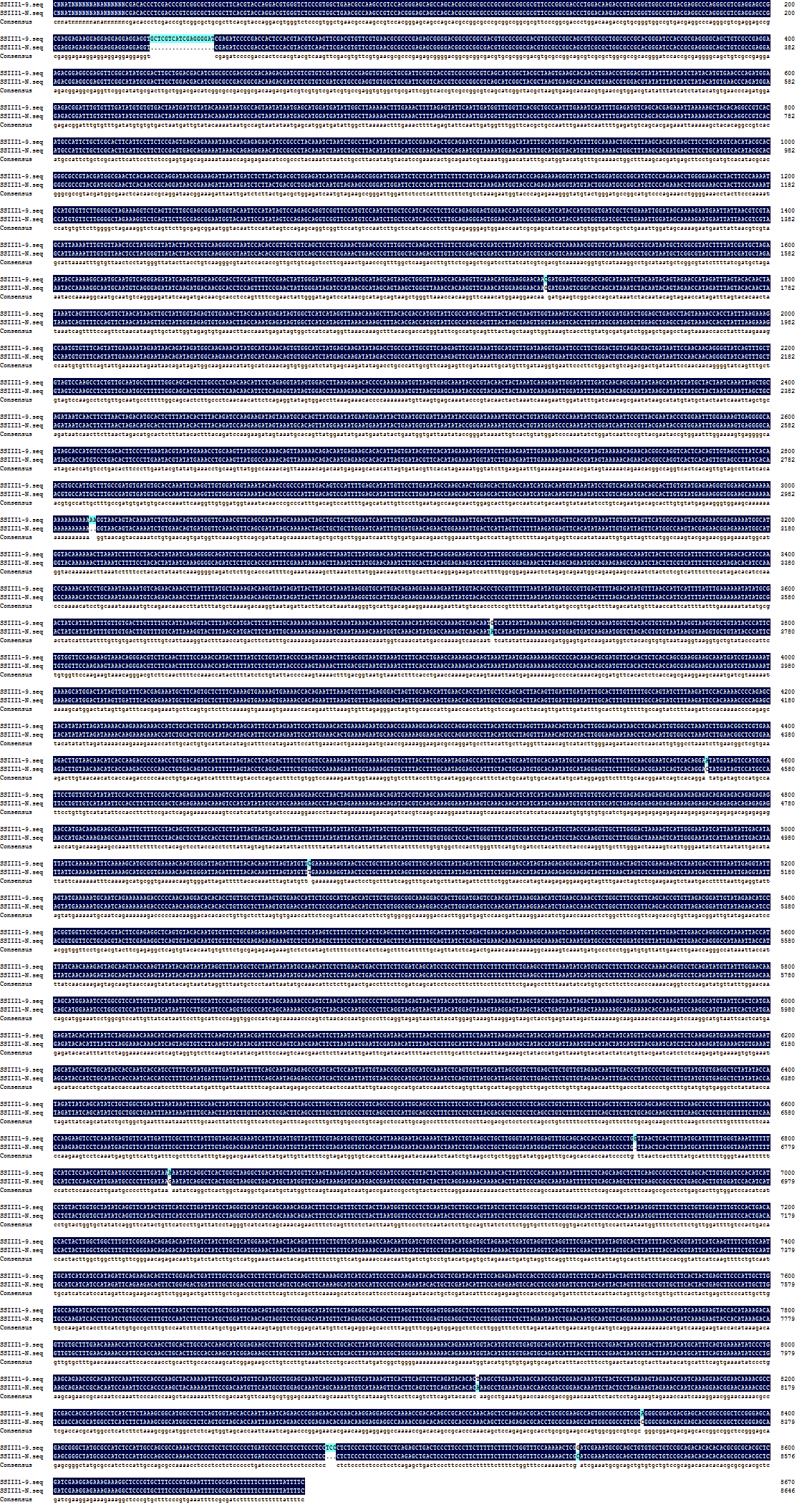


Result of protein sequence alignment

1. ***SSIII–2*** (<http://www.gramene.org/>, *LOC_Os08g09230* and *BGIOSGA028122*)

Result of DNA sequence alignment

Result of protein sequence alignment

1. ***SSIV–1*** (<http://www.gramene.org/>, *LOC_Os01g52250* and *BGIOSGA000900*)

Result of DNA sequence alignment

Result of protein sequence alignment

1. ***SSIV–2*** (<http://www.gramene.org/>, *LOC_Os05g45720* and *BGIOSGA020250*)

Result of DNA sequence alignment

Result of protein sequence alignment

1. ***SBE1*** (<http://www.gramene.org/>, *LOC_Os06g26234* and *BGIOSGA040463*)

Result of DNA sequence alignment

Result of protein sequence alignment

1. ***SBE3*** (<http://www.gramene.org/>, *LOC_Os02g32660* and *BGIOSGA006344*)

Result of DNA sequence alignment

Result of protein sequence alignment

1. ***SBE4*** ([http://www.gramene.org/, *LOC_Os04g33460*](http://www.gramene.org/,%20LOC_Os04g33460) and *BGIOSGA015140*)

Result of DNA sequence alignment

Result of protein sequence alignment

1. ***ISA*** ([http://www.gramene.org/,](http://www.gramene.org/,%20LOC_Os04g33460) *LOC_Os08g40930* and *BGIOSGA026650*)

Result of DNA sequence alignment

Result of protein sequence alignment

**17. *PUL*** ([http://www.gramene.org/,](http://www.gramene.org/,%20LOC_Os04g33460) *Os04G0164900* and *BGIOSGA015875*)

Result of DNA sequence alignment

Result of protein sequence alignment
